# Obinutuzumab-induced acute thrombocytopenia: a case report and literature review

**DOI:** 10.3389/fonc.2024.1509567

**Published:** 2024-12-17

**Authors:** Xuelin Dou, Kongyang Li, Jin Lu

**Affiliations:** ^1^ Department of Hematology, Peking University People’s Hospital, National Clinical Research Center for Hematologic Diseases, Peking University Institute of Hematology, Beijing, China; ^2^ Department of Hematology, People’s Hospital of Yangjiang, Yangjiang, China

**Keywords:** CD20 antibodies, lympho proliferative disorder, adverse (side) effects, obinutuzumab (GA101), acute thrombocytopenia

## Abstract

**Background:**

Obinutuzumab, a humanized type II anti-CD20 monoclonal antibody, is widely used in the treatment of B-cell lymphomas. Thrombocytopenia typically occurs 1 to 2 weeks after administration. In rare cases, obinutuzumab can induce severe acute thrombocytopenia within days of infusion, a condition known as “obinutuzumab-induced acute thrombocytopenia (OIAT).” Rituximab, a chimeric type I anti-CD20 monoclonal antibody, is also known to cause “rituximab-induced acute thrombocytopenia (RIAT).” This report presents a case of OIAT, with subsequent treatment switched to rituximab, which did not result in thrombocytopenia recurrence.

**Case Presentation:**

A 38-year-old female patient with a 2-year history of lymphadenopathy was diagnosed with follicular lymphoma (Grade I-II). She was treated with obinutuzumab combined with bendamustine. Following the first administration of obinutuzumab, her platelet count dropped to 37×10⁹/L within 2 days and further declined to 27×10⁹/L on the fourth day without bleeding symptoms. The platelet count recovered by day 8. After a second obinutuzumab infusion, the platelet count again dropped to 15×10⁹/L within 1 day. Platelet transfusion was effective, and the count eventually recovered to 95×10⁹/L by day 29. No further acute thrombocytopenia occurred after switching to rituximab.

**Conclusion:**

OIAT is a rare but serious adverse effect of obinutuzumab. This case highlights the importance of early recognition and monitoring of platelet counts in patients receiving obinutuzumab. The findings in our case, along with those in the literature, suggest that switching to rituximab or extending the interval before obinutuzumab re-administration can reduce the risk of recurrent thrombocytopenia. Further research is needed to elucidate the underlying mechanisms and establish treatment guidelines for OIAT.

## Introduction

Obinutuzumab is a novel humanized type II anti-CD20 monoclonal antibody that has undergone glycosylation modification in the Fc region, enhancing its affinity for Fc receptors on immune effector cells. This modification results in stronger antibody-dependent cellular cytotoxicity (ADCC) and phagocytosis, as well as more potent direct B-cell killing compared to rituximab, a first-generation chimeric anti-CD20 monoclonal antibody (type I) (1, 2). Obinutuzumab was approved by the National Medical Products Administration (NMPA) in June 2021 for use in combination with chemotherapy for the treatment of previously untreated follicular lymphoma in adult patients and for monotherapy in those who have achieved at least a partial response (3). It has also been approved by the US Food and Drug Administration (FDA) for use in relapsed/refractory follicular lymphoma and previously untreated chronic lymphocytic leukemia (4).

Common serious adverse reactions to obinutuzumab include infusion-related reactions, infections, neutropenia, and thrombocytopenia (5–7). Thrombocytopenia typically occurs within 1-2 weeks of administration. However, obinutuzumab-induced acute thrombocytopenia (OIAT), occurring within days of infusion, is a rare and severe adverse reaction. This report describes a case of OIAT in a patient treated for follicular lymphoma and reviews related literature.

## Case presentation

A 38-year-old female patient was admitted in July 2024 for evaluation of a 2-year history of lymphadenopathy. In October 2021, she noted enlargement of cervical lymph nodes, followed by axillary and inguinal lymph node involvement in October 2022. A PET-CT scan on November 10, 2022, revealed multiple hypermetabolic lymph nodes, with the largest located in the porta hepatis, measuring 3.2×2.9 cm with an SUVmax of 6.7. Splenomegaly with increased metabolism (SUVmax 4.1) was also noted.

A core needle biopsy of the right inguinal lymph node revealed non-Hodgkin follicular lymphoma (Grade I-II) with active tumor follicle growth. The patient was initially observed with no indication for immediate treatment. In June 2024, she presented with chest tightness, and a CT scan showed multiple enlarged lymph nodes in the neck, supraclavicular region, mediastinum, and bilateral axillary regions. Pleural effusion with compressive atelectasis of the right lower lung was also noted. Laboratory tests revealed normal blood counts and biochemical parameters. Bone marrow biopsy and flow cytometry confirmed the diagnosis of B-cell non-Hodgkin lymphoma, with a immunophenotype supporting follicular lymphoma. Patient was diagnosed with follicular Lymphoma with grade: I-II, Ann Arbor Stage IVA, with FLIPI-1 Score of 2, and a FLIPI-2 Score of 1.

**Figure 1:**
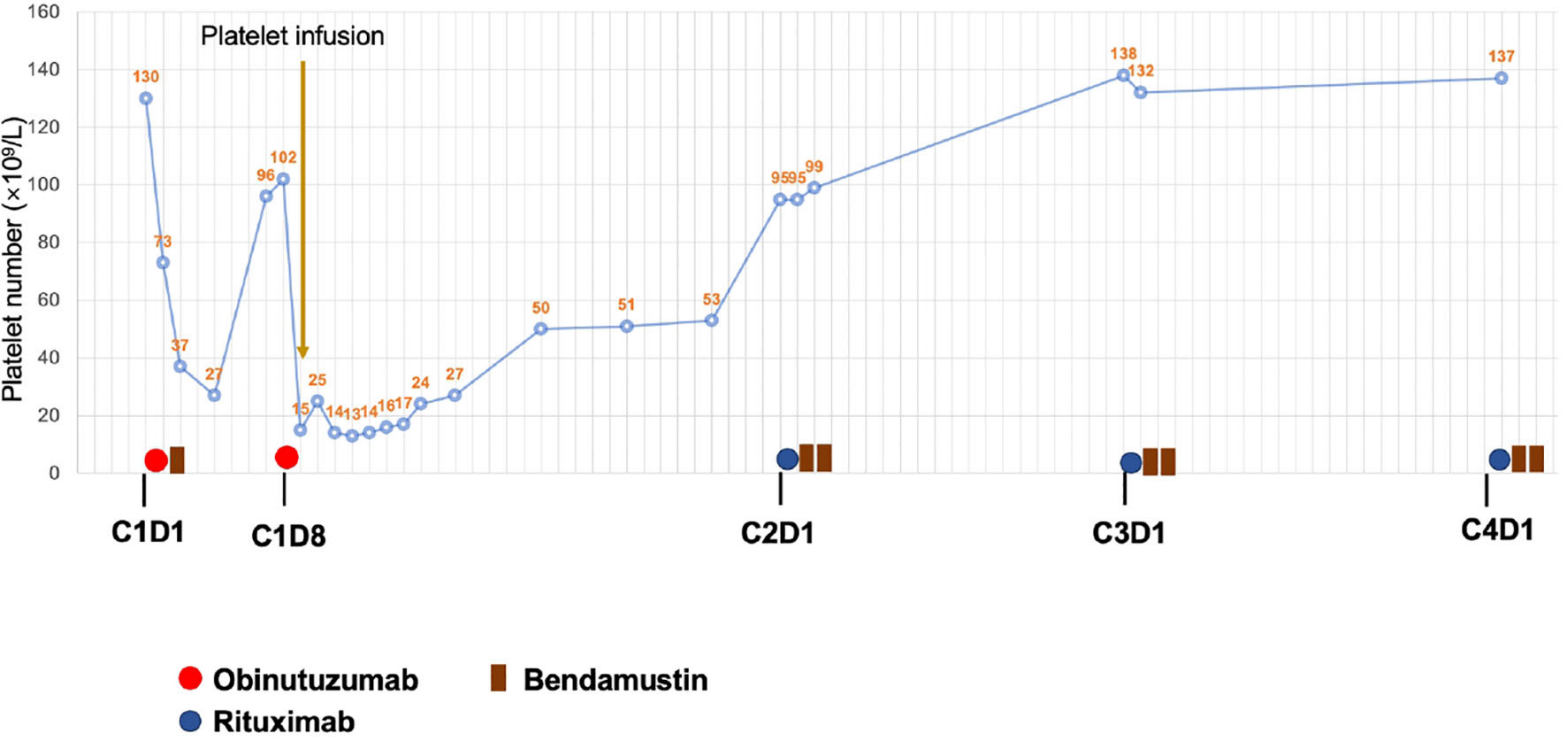
Changes of platelet number and treatment overview.

The patient was started on chemotherapy with obinutuzumab (1000 mg) and bendamustine (120 mg, i.e., 70mg/m2) on July 7, 2024 as C1D1 treatment. Notably, there were no infusion-related reactions; the patient did not experience fever, chills, chest distress, or other discomforts. However, her C-reactive protein (CRP) was elevated at 53 mg/L (normal range: 0–10 mg/L) at the second day after treatment (C1D2), compared to baseline, and her D-dimer level increased to 6715 ng/mL (normal range: 0–243 ng/mL) from a baseline of 407 ng/mL at C1D2. Two days after the first infusion, her platelet count dropped to 37×10⁹/L, further declining to a nadir of 27×10⁹/L on day C1D4, without bleeding symptoms. Platelet recovery was noted on day C1D8. Following the second infusion of obinutuzumab on C1D8, the platelet count again dropped to 15×10⁹/L within 1 day, requiring platelet transfusion. Both CRP and D-dimer levels returned to baseline level before and after the second obinutuzumab infusion. The platelet count recovered to 95×10⁹/L by day 29. The patient was subsequently switched to rituximab and bendamustine for further treatment, and no recurrence of thrombocytopenia occurred (**Figure 1**). As of November 2024, the patient has successfully completed five more cycles of bendamustine and rituximab (BR) therapy, achieving a partial response (PR) as evaluated by contrast-enhanced CT scans in accordance with the Lugano 2014 criteria. The patient is scheduled to receive two additional cycles of rituximab, followed by maintenance therapy with rituximab administered every two months for up to two years.

## Discussion and literature review

*In vitro* studies have shown that obinutuzumab has a stronger B-cell killing effect (1). However, the incidence of adverse drug reactions, including thrombocytopenia, infusion-related reactions, neutropenia, and infections, is higher with obinutuzumab compared to rituximab (5). Thrombocytopenia is a common serious adverse event associated with obinutuzumab, and similar adverse effects are observed with the first-generation chimeric type I anti-CD20 monoclonal antibody, rituximab. A meta-analysis comparing the efficacy and safety of new anti-CD20 monoclonal antibodies with rituximab across four clinical trials (including the GAINED, GALLIUM, GAUSS, and GOYA studies) concluded that obinutuzumab was associated with higher rates of total adverse events, grade 3-5 adverse events, infusion-related reactions, neutropenia, grade 3-5 thrombocytopenia, and grade 3-5 infections compared to rituximab. Specifically, the incidence of grade 3-5 thrombocytopenia in the obinutuzumab group was significantly higher than in the rituximab group (OR 2.67, 95% CI 1.69–4.23) (5). In the GALLIUM trial, which focused on the treatment of follicular lymphoma, 26 out of 595 patients (11.4%) in the obinutuzumab-chemotherapy group developed thrombocytopenia, compared to 16 out of 597 patients (7.5%) in the rituximab-chemotherapy group. The rates of grade 3-5 thrombocytopenia were 6.1% and 2.7%, respectively (6). In the GAIA-CLL13 study on chronic lymphocytic leukemia, the incidence of grade ≥3 thrombocytopenia was 14.9% (34/228) in the venetoclax-obinutuzumab group, compared to 3.4% (8/237) in the venetoclax-rituximab group (7). However, these studies did not emphasize the timing of severe thrombocytopenia, making it difficult to compare the incidence of OIAT (obinutuzumab-induced acute thrombocytopenia) with RIAT (rituximab-induced acute thrombocytopenia).

Currently, more than 30 cases of RIAT have been reported in the literature, whereas reports of OIAT are rare and mainly consist of case reports (8). A PubMed search using the keywords “obinutuzumab” and “acute thrombocytopenia” retrieved 9 OIAT cases (9–14). **Table 1** presents the basic information of the related case reports and the case from this study. The first case of OIAT was described by Walter et al. in 2016, involving a 68-year-old female chronic lymphocytic leukemia patient who developed OIAT 50 hours after receiving obinutuzumab and venetoclax treatment (9). Including the current case, a total of 10 OIAT cases have been reported, with 7 female and 2 male patients, and one case where the gender was not mentioned. The median age was 56 years. Treatment regimens included obinutuzumab monotherapy in 1 case, obinutuzumab combined chemotherapy in 8 cases, and obinutuzumab with bispecific antibodies in 1 case. The median time to OIAT onset was 2 days, with 9 cases occurring during the initial obinutuzumab treatment and 1 during maintenance therapy. Treatments included platelet transfusion in 8 cases, platelet transfusion with intravenous immunoglobulin in 1 case, platelet transfusion with a thrombopoietin receptor agonist (romiplostim) in 1 case, and clinical observation for the first episode followed by platelet transfusion for recurrent OIAT in 1 case. Six patients had complete platelet recovery within a median of 8 days (range 6-22), while 3 patients partially recovered to >50×10⁹/L within 5 days (range 4-8). Only 1 patient required platelet transfusions 23 days after treatment. In terms of subsequent treatments, 2 patients switched to rituximab without recurrence of severe thrombocytopenia, while 8 patients continued obinutuzumab; 4 of these patients did not experience OIAT again, but the other 4 cases had recurrent OIAT. One patient with recurrent OIAT switched to rituximab, with no further episodes of thrombocytopenia. It should be noted that the timing of re-administration of obinutuzumab appears to play a significant role in preventing recurrent OIAT. Yılmaz et al. reported three cases in which obinutuzumab was re-administered only at the time of the second cycle (rather than on Day 8 or Day 15 of the first cycle), and none of these cases experienced recurrent OIAT (14). This observation suggests that extending the interval between doses may reduce the risk of recurrence. Our case did not adopt this approach, but switching to rituximab also successfully prevented further episodes of thrombocytopenia.

**Table 1:** Clinical characteristics and outcomes of 9 cases reported in the literature and the current OIAT case.

No.	Source	Disease	Gender	Age (Years)	Bone Marrow Involvement	Treatment Plan	Pre-Treatment Platelet Count (10^9/L)	Lowest Platelet Count (10^9/L)	OIAT Occurrence Time	Treatment Method	Platelet Recovery	Re-infused with Obinutuzumab/Recurrence
1	Walter et al. (9)	CLL	Female	68	Yes	G+Ven	150	40	50h	Platelet transfusion	+7d returned to normal	Re-infused/No recurrence
2	Sakai et al. (10)	FL	Female	64	NR	G	160	21	6h	Platelet transfusion	+10d returned to normal	Re-infused/Multiple recurrence during the following treatment
3	Haage et al. (11)	FL	Female	56	Yes	G+B	245	4	1d	Platelet transfusion, IVIG	+6d returned to normal	Switched to R/No recurrence
4	Mechelfekh et al. (12)	FL	Female	74	No	G+CVP	376	3	1d	Platelet transfusion, Romiplostim	+22d returned to normal	Switched to R/No recurrence
5	Mechelfekh et al. (12)	MCL	Female	44	Yes	G+Glo	76	3	1d	Platelet transfusion	+8d >50×10^9/L, +21d highest recovery to >90×10^9/L	Re-infused/Recurrence after the first course
6	Ng, J.Y. et al. (13)	CLL	Female	83	Yes	G+Ven	104	4	48h	Platelet transfusion	+8d returned to normal	Re-infused/Recurrence after the first course
7	Yılmaz et al. (14)	DLBCL	Female	81	No	G+Len	144	33	2d	Platelet transfusion	+5d > 90×10^9/L	Re-infused/No recurrence
8	Yılmaz et al. (14)	FL	Male	47	Yes	G+ICE	111	23	2d	Platelet transfusion	+4d > 50×10^9/L	Re-infused/No recurrence
9	Yılmaz et al. (14)	FL	Male	41	NR	G+ICE	112	13	4d	Platelet transfusion	Still dependent on transfusion at +23d	Re-infused/No recurrence
10	Current Case (First course)	FL	Female	38	Yes	G+B	130	27	2d	Clinical observation	+8d returned to normal	Re-infused/Recurrence
11	Current Case (Second course)	FL	Female	38	Yes	G+B	192	15	1d	Platelet transfusion	+29d highest recovery to 95×10^9/L	Switched to R/No recurrence

NR, not reported.

The mechanism underlying anti-CD20 monoclonal antibody-induced acute thrombocytopenia remains unclear. While the mechanism for obinutuzumab-induced thrombocytopenia is not well understood, more research has been conducted on RIAT. Huang AJ et al. reported 1 case and summarized an additional 31 RIAT cases, proposing five potential mechanisms: 1, platelet surface expression of FcγRIIa (CD32a) may mediate platelet degradation when rituximab-bound lymphoma cells interact with platelets through Fc receptors; 2, rituximab activates the immune system through its interaction with tumor cells, leading to consumptive coagulopathy and platelet depletion; 3, complement activation following rituximab infusion may directly destroy platelets or promote the release of cytokines such as TNF-α, which mediate thrombocytopenia; 4, soluble CD20 antigen in the patient’s serum may cause an antigen-antibody reaction, leading to immune-mediated cell lysis; 5, lymphoma infiltration of the spleen may alter endothelial structures, and exposure of the endothelium following rituximab-mediated tumor cell clearance may trigger platelet activation and aggregation (8). These mechanisms may also apply to OIAT, with the enhanced immune effects of obinutuzumab (due to its glycosylated Fc region) possibly contributing to its higher incidence of thrombocytopenia compared to rituximab. Additionally, basic research has demonstrated that PD-L1 can be transferred from non-small cell lung cancer cells to platelets (15). Haage et al. hypothesized that in patients with lymphoma involving the bone marrow, CD20 could be transferred to platelets, causing them to express CD20 and leading to OIAT (11). In chronic lymphocytic leukemia patients, pro-inflammatory cytokines such as IL-6 and IL-8 are rapidly released following the initial obinutuzumab infusion, particularly in patients with a baseline absolute lymphocyte count (ALC) ≥50×10⁹/L. These patients are more likely to experience cytopenias during the first treatment cycle (16). This hypothesis may explain why thrombocytopenia often occurs during the initial use of anti-CD20 monoclonal antibodies, particularly in patients with high tumor burden or bone marrow involvement.

Due to the limited number of reported cases, there is no standardized treatment protocol for OIAT. Treatment must be individualized based on the patient’s condition. Reported treatments include platelet transfusion, thrombopoietin receptor agonists, and intravenous immunoglobulin. Among the 10 cases reviewed, all patients received platelet transfusions, with 8 showing platelet recovery after transfusion. However, 2 cases exhibited transfusion refractoriness, which was resolved with thrombopoietin receptor agonists (romiplostim) or high-dose intravenous immunoglobulin. Given the possible immune mechanism underlying OIAT, treatment strategies for immune thrombocytopenia (ITP) may also be applied, including glucocorticoids, intravenous immunoglobulin, and thrombopoietin receptor agonists.

Early identification of this serious complication is crucial. Due to the lack of routine monitoring of platelet counts in patients receiving obinutuzumab, OIAT diagnosis may be delayed. Most reported cases of OIAT occurred within 1-3 days after obinutuzumab infusion. In a case reported by Sakai et al., platelet monitoring detected thrombocytopenia as early as 1 hour after obinutuzumab infusion (10). Earlier monitoring in other OIAT cases may have revealed similar patterns. We recommend routine platelet monitoring within 24 hours of obinutuzumab infusion, with closer monitoring if thrombocytopenia is detected, including 1-hour pre- and post-transfusion platelet counts to assess transfusion efficacy.

Switching from obinutuzumab to rituximab is another strategy to prevent recurrent OIAT. In this case and others, patients who developed OIAT and were subsequently switched to rituximab did not experience further thrombocytopenia. However, the potential for recurrent OIAT in patients who continue obinutuzumab remains uncertain. In this report, the patient experienced OIAT after the first obinutuzumab infusion, with more severe thrombocytopenia after the second infusion (15×10⁹/L vs. 27×10⁹/L) and a longer recovery time (29 days vs. 8 days). Switching to rituximab prevented further thrombocytopenia. However, given the limited number of cases, it remains unclear whether continued use of obinutuzumab after OIAT will always result in recurrent thrombocytopenia. Thorough communication with the patient is essential to weigh the risks of OIAT against the benefits of continuing obinutuzumab therapy. In cases of recurrent OIAT, switching to rituximab should be considered, along with close platelet monitoring.

In conclusion, the exact mechanism of OIAT remains unclear and requires further research. Increased awareness of OIAT is essential, along with enhanced platelet monitoring in patients receiving obinutuzumab to allow for early detection and timely intervention. Treatment strategies should be based on the severity of thrombocytopenia and may include platelet transfusion and switching from obinutuzumab to rituximab.

## Conclusion

OIAT is a rare but serious complication of obinutuzumab therapy. Early recognition and monitoring of platelet counts in patients receiving obinutuzumab are crucial for preventing severe outcomes. The findings in our case, along with those in the literature, suggest that switching to rituximab or extending the interval before obinutuzumab re-administration can reduce the risk of recurrent thrombocytopenia. Further studies are needed to clarify the underlying mechanisms of OIAT and establish standardized management guidelines.

## Data Availability

The raw data supporting the conclusions of this article will be made available by the authors, without undue reservation.
